# The Effect of Organic Vegetable Mixed Juice on Blood Circulation and Intestine Flora: Randomized, Double-Blinded, Placebo-Controlled Clinical Trial

**DOI:** 10.3390/diseases12090223

**Published:** 2024-09-23

**Authors:** Yun-Ha Lee, Jae-Ho Lee, Soo-Min Jeon, Il-Kyu Park, Hyun-Bin Jang, Soo-A Kim, Soo-Dong Park, Jae-Jung Shim, Seong-Soo Hong, Jae-Hwan Lee

**Affiliations:** 1 R&BD Center, Hy Co., Ltd., 22, Giheungdanji-ro 24beon-gil, Giheung-gu, Yongin-si 17086, Republic of Korea; yunha1006@hy.co.kr (Y.-H.L.); jhlee@hy.co.kr (J.-H.L.); 10003273@hy.co.kr (S.-M.J.); ikpark@hy.co.kr (I.-K.P.); hbjang@hy.co.kr (H.-B.J.); freebi7@hy.co.kr (S.-A.K.); soodpark@hy.co.kr (S.-D.P.); jjshim@hy.co.kr (J.-J.S.); 2 Department of Gastroenterology, Vievis Namuh Hospital, 627, Nonhyeon-ro, Gangnam-gu, Seoul 06117, Republic of Korea

**Keywords:** vegetable juice, lipid profile, antioxidants, blood circulation, gut microbiome

## Abstract

Epidemiological evidence suggests that fruit and vegetable intake significantly positively affects cardiovascular health. Since vegetable juice is more accessible than raw vegetables, it attracts attention as a health functional food for circulatory diseases. Therefore, this study measured blood lipids, antioxidants, blood circulation indicators, and changes in the microbiome to confirm the effect of organic vegetable mixed juice (OVJ) on improving blood circulation. This 4-week, randomized, double-blinded, placebo-controlled study involved adult men and women with borderline total cholesterol (TC) and low-density lipoprotein (LDL) levels. As a result, blood lipid profile indicators, such as TC, triglycerides, LDL cholesterol, and apolipoprotein B, decreased (*p* < 0.05) in the OVJ group compared with those in the placebo group. Additionally, the antioxidant biomarker superoxide dismutase increased (*p* < 0.05). In contrast, systolic and diastolic blood viscosities, as blood circulation-related biomarkers, decreased (*p* < 0.05) in the OVJ group compared with those in the placebo group. After the intervention, a fecal microbiome analysis confirmed differences due to changes in the intestinal microbiome composition between the OVJ and placebo groups. In conclusion, our research results confirmed that consuming OVJ improves blood circulation by affecting the blood lipid profile, antioxidant enzymes, and microbiome changes.

## 1. Introduction

Circulatory diseases such as cardiovascular diseases are emerging as an essential problem in modern people due to changes in dietary habits [1]. A balanced diet with appropriate fruits and vegetables can support a healthy heart and circulatory system. Epidemiological evidence suggests that fruit and vegetable intake significantly positively affects cardiovascular health [2,3,4]. Since vegetable juice is more accessible than raw vegetables, it attracts attention as a healthy, functional food for circulatory diseases [1]. Various studies have shown that juice is part of a balanced diet that significantly reduces the risk of many diseases, such as cancer and cardiovascular disease [5,6,7].

Vegetables and fruits are widely recommended because they are rich sources of numerous active ingredients that support overall health and reduce the risk of chronic diseases, such as cardiovascular disease [8]. In particular, they contain essential elements, such as potassium, antioxidants, vitamins, and folic acid, that may help lower blood pressure, a significant risk factor for cardiovascular disease [9]. Also, fruits and vegetables have beneficial effects on the human body related to anti-nutrients, such as low-molecular-weight antioxidants used to improve quality of life and prevent disease [10]. Biologically active compounds in plant foods, such as redox-active antioxidants (vitamins C and E, polyphenols, carotenoids, tocopherols, and glutathione (GSH)) and enzymes with antioxidant activity (superoxide dismutase (SOD) and catalase (CAT)), potentially regulate multiple processes during disease development [11].

Many previous studies have reported that consuming vegetable or fruit juice can improve human health. For example, one study looked at the association between orange juice consumption and cardiovascular disease in women and men with cardiovascular disease. As a result, total cholesterol (TC), apo B, low-density lipoprotein-cholesterol (LDL-C), and the low-density lipoprotein/high-density lipoprotein (LDL/HDL) ratio were all significantly lower in the orange juice consumption group [12]. In another study, lycopene and tomato products reduced plasma total cholesterol and LDL cholesterol and increased high-density lipoprotein cholesterol in animal experiments. Consistently with these experimental results, a clinical trial also showed that dietary supplements containing lycopene and/or tomato products reduced plasma LDL cholesterol [13]. And there was a study that confirmed that consuming *Lycium barbarum* juice was effective in antioxidant activity by increasing antioxidant biomarkers SOD and GSH-Px and decreasing MDA (malondialdehyde) [14]. There have been many studies on vegetable or fruit juice intake. Still, all of them used juice made from a single type of fruit or vegetable as the main ingredient, and as far as we know, no clinical study has been conducted on juice mixed with various vegetables. In addition, these studies did not measure multiple indicators simultaneously. Instead, they measured individual indicators such as blood lipids, antioxidants, and blood circulation improvement to confirm the effect on one subject.

Manifold studies have suggested that gut microbiota are critical to human health. Their diversity and composition have significantly been implicated in the development of disorders such as irritable bowel syndrome, cardiovascular disease, diabetes, and obesity [15,16,17]. Diet is reportedly one of the most critical factors affecting gut microbiota composition [17]. The effects of vegetable and fruit intake on the gut microbiome have been explored [18,19,20,21,22]. The consumption of fruits and vegetables, which are sources of polyphenols, oligosaccharides, fiber, and environmental microbiota, has a potentially beneficial effect on the functional diversity of the gut microbiome, including microbes harboring genes involved in the synthesis of vitamins and short-chain fatty acids (SCFAs) [18,19]. 

The most abundant gut microorganism-generated SCFAs, such as butyrate, acetate, and propionate, have widespread effects on host physiology [23]. Among the plant components that affect the intestinal microbial community, the most extensively studied are plant-derived dietary fiber (non-digestible polysaccharides) and polyphenols. Fiber is an essential component of the human diet and is known to possess health benefits, including reduced blood sugar and cholesterol and improved glycemic response [24]. Various studies have recently confirmed the association of vegetable and fruit consumption with cardiovascular disease, antioxidant effects, and intestinal microorganisms [25]. Substantial evidence suggests that intestinal microbiota are closely related to the cardiovascular system through functions such as the fermentation of dietary fiber, production of SCFAs, and absorption of phytochemicals in the large intestine [26,27]. However, as far as we know, there have been no studies confirming the efficacy in the human body using an approach based on changes in the composition of the microbiome and functional prediction by consuming vegetable juice mixed with various vegetables.

Therefore, this clinical trial aimed to determine changes in blood lipid levels, antioxidant levels, and intestinal microbiome after consuming organic vegetable mixed juice (OVJ). We also conducted research to determine whether changes in these indicators could help improve blood circulation.

## 2. Materials and Methods

### 2.1. Test Materials

The test juice used in this study was an organic vegetable mixed juice provided by Hy Co., Ltd. The main ingredients of the test juice are organic tomato paste and organic carrot concentrate, and the rest consists of organic green vegetable mixed juice (lettuce, celery, spinach, bok choy, endive, mugwort, cabbage, kale, cauliflower, etc.). Participants consumed two bottles (100 mL per bottle) at a time (200 mL/day) 30 min after meals, once a day for 4 weeks. The control juice possessed a similar color and taste to the test juice but contained no vegetables or fruits. Nutrient and functional ingredient contents are presented in Table 1 and Table 2.

### 2.2. Study Participants and Inclusion/Exclusion Criteria

In this study, 60 otherwise healthy male and female participants were recruited from Vievis Namuh Hospital, Seoul, Republic of Korea. They were aged >19 and ≤75 years. Those who agreed to participate in this clinical trial before the study initiation and completed a written informed consent form participated. Adult men and women with borderline TC and LDL levels and not having a diagnosed disease were included in the study. The exclusion criteria were as follows: (1) disorders in platelet function or coagulation; (2) taking oral or injectable diabetes medications such as anticoagulants, warfarin, antiplatelet agents (e.g., aspirin and clopidogrel), and calcium antagonists or hormonal agents such as dexamethasone, betamethasone, somatropin, and lutropin alfa within the preceding 2 weeks; (3) body mass index (BMI) < 18.5 or >30 kg/m^2^; (4) taking medicines or health-functional foods related to platelet function, blood circulation improvement, or hyperlipidemia within 4 weeks of participating in the study; (5) consuming blood flow-related health-functional foods, such as red ginseng, ginseng, natto, guava leaf extract, and indigestible maltodextrin, within 2 weeks from the date of the checkup visit.

### 2.3. Study Design

This 4-week randomized, double-blinded, placebo-controlled study on healthy male and female Koreans was approved by the Ethics Board Committee of Vievis Namuh Hospital (IRB No. VNIRB-202411). Participants were notified through screening that they had satisfied the selection criteria and were scheduled for the next visit via phone. Written informed consent was obtained from all participants before enrollment. Assessments were conducted at initial visit for screening, and at weeks 0 and 4 after randomization. At the baseline (second) visit, participants were randomly assigned to test and placebo (control juice) groups. Randomization lists were computer-generated by a statistician. Participants and investigators were blinded to the intervention assignment until the end of the study. According to published human dietary intervention study guidelines, the required number of participants was determined using a power calculation. A trained researcher monitored compliance by quantifying the remaining juice collected from participants at the final visit. Before and after the intervention, both groups were evaluated for various parameters (anthropometric variables, biochemical indicators, and vital signs). Participants were also examined for any adverse effects during the intervention.

### 2.4. Outcome Measures

Lipid metabolism indicators (total cholesterol [TC], triglycerides [TG], high-density lipoprotein cholesterol [HDL-C], low-density lipoprotein cholesterol [LDL-C], very low-density lipoprotein [VLDL], and apolipoprotein B [ApoB]) at 4 weeks were compared to those at baseline. Blood samples were collected after 12 h of overnight fasting. Serum concentrations of lipid profiles, including TG, TC, HDL-C, LDL-C, VLDL, and ApoB, were measured via colorimetric methods using appropriate commercial kits. In addition, the same antioxidant indicators, namely catalase (CAT), malondialdehyde (MDA), superoxide dismutase (SOD), glutathione (GSH), oxidized LDL (Ox LDL), blood circulation indicators, endothelial NOS (e-NOS), thromboxane B2, changes in systolic and diastolic blood viscosity prothrombin time (PT), and activated partial thromboplastin time (aPTT), were also evaluated. Antioxidants (CAT, MDA, SOD, GSH, and Ox LDL) and blood circulation-related indicators (e-NOS, thromboxane B2) were measured via the enzyme-linked immunosorbent assay using dedicated kits. Systolic and diastolic blood viscosities were assessed using a scanning capillary tube viscometer (Hemovister, Pharmode Inc., Seoul, Republic of Korea). PT and aPTT were assayed on a fully automatic Stago Coagulometer STA^®^ Compact Max (Diagnostica Stago, Paris, France) using STA-Neoplasitne^®^ Cl Plus and STA-PTT A (Diagnostica Stago, Asnieres, France).

### 2.5. Safety

Participants had their biochemical parameters and vital signs (blood pressure, pulse rate) assessed before, and after 4 weeks of the intervention. Systolic and diastolic blood pressure were assessed after a rest period (20 min). Vital signs were measured twice on the left arm using an automatic blood pressure monitor, and the average of the two measurements was used. Before and after 4 weeks of the intervention, participants’ biochemical parameters were evaluated using a Siemens ADVIA^®^ 1800 instrument (Siemens, München, Germany). Hematological (red blood cells, hemoglobin, hematocrit, white blood cells, platelets, red cell distribution width, segmented neutrophils, lymphocytes, monocytes, eosinophils, and basophils), blood chemical (aspartate transaminase, alanine aminotransferase, creatine phosphokinase, total protein, albumin, and glucose), and urine (total bilirubin, urine color, turbidity, occult blood, WBC stick, specific gravity, pH, glucose, ketone, and bilirubin) tests were performed.

### 2.6. Statistical Analysis

Data are presented as the mean ± standard error of the mean (SEM). For continuous variables, normality was determined using the Shapiro–Wilk test. Normally distributed variables were tested for significance using the two-sample *t*-test, while non-normally distributed variables were tested for significance using the Mann–Whitney U-test. Within-group comparisons were performed using paired *t*-tests. Statistical significance was set at *p* < 0.05.

### 2.7. Fecal Microbiome Analysis

#### 2.7.1. Sample Handling and Collection

Stool samples were collected from the study participants pre- (week 0) and post-intervention (week 4). Feces were acquired using a stool paper and stool container, transported to the laboratory in an insulated bag containing an ice pack, and subsequently frozen and stored at −80 °C until use. Stool samples from all subjects were individually sequenced after DNA extraction to digitize the data. According to the manufacturer’s instructions, DNA was isolated in several batches to reach the desired amount using the MoBio PowerSoil^®^ DNA Isolation Kit (Qiagen, Hilden, Germany). DNA samples were carefully quantified using a NanoDrop™ Spectrophotometer (Thermo Scientific, Waltham, MA, USA), and A260/A280 ratios were measured to confirm a high-purity DNA yield. They were frozen and stored at −20 °C until use.

#### 2.7.2. 16S rRNA Gene Amplicon Sequencing

Sequencing libraries were prepared by amplifying the V3 and V4 regions according to the Illumina 16S Metagenomic Sequencing Library protocol. The input gDNA (2 ng) was polymerase chain reaction (PCR)-amplified using 5× reaction buffer, 1 mM deoxyribonucleotide triphosphate mix, 500 nM of each universal forward/reverse PCR primer, and Herculase II fusion DNA polymerase (Agilent Technologies, Santa Clara, CA, USA). Thermal cycling for the first PCR step included 3 min denaturation at 95 °C; 25 cycles of 30 s at 95 °C, 30 s at 55 °C; and 30 s at 72 °C; and a 5 min final extension at 72 °C. The following universal primer pair with Illumina adapter overhang sequences was used for the first amplifications: V3-F (50-TCGTCGGCAGCGTCAGATGTGTATAAGAGACAGCCTACGGGNGGCWGCAG-30) and V4-R (50-GTCTCGTGGGCTCGGAGATGTGTATAAGAGACAGGACTACHVGGGTATCTAATCC-30). The PCR product from the first step was purified using AMPure beads (Agencourt Bioscience, Beverly, MA, USA). Following purification, 2 µL of the PCR product from the first step was PCR-amplified for final library construction using the Nextera™ XT Indexed Primer (Illumina, San Diego, CA, USA). Thermal cycling for the second PCR step was performed as described for the first step, albeit with 10 instead of 25 cycles. The resulting PCR product was purified with AMPure beads, quantified using quantitative PCR (qPCR) according to the qPCR Quantification Protocol Guide (KAPA Library Quantification Kits for Illumina sequencing platforms), and qualified using a TapeStation D1000 ScreenTape (Agilent Technologies, Waldbronn, Germany).

#### 2.7.3. Analysis of Operational Taxonomic Units (OTUs) Microbiome Analysis

A rarefaction curve was constructed to determine the saturation of amplicon sequence variant sequencing. Biological diversity was evaluated based on alpha and beta diversity indices using the physeq package in R software (version 4.2.1) [28]. Shannon and Simpson indices were used to determine species richness, and Bray–Curtis dissimilarities were used to indicate differences between the two groups. Statistical *p* values were obtained using the Adonis test. To detect differentially abundant microbial communities, a negative binomial-based generalized linear model was formulated using the edgeR package with the Trimmed Mean of M-value normalization method to consider the diverse read generation [29]. Phylogenetic Investigation of Communities by Reconstruction of Unobserved States (PICRUSt2) and Kyoto Encyclopedia of Genes and Genomes (KEGG) databases were used to compare functional profiles [30]. A Spearman’s rank correlation analysis was used to infer correlations between biological outcomes and bacterial operational taxonomic units (OTUs).

## 3. Results

### 3.1. Enrollment (Participant Baseline Characteristics)

**Figure 1:**
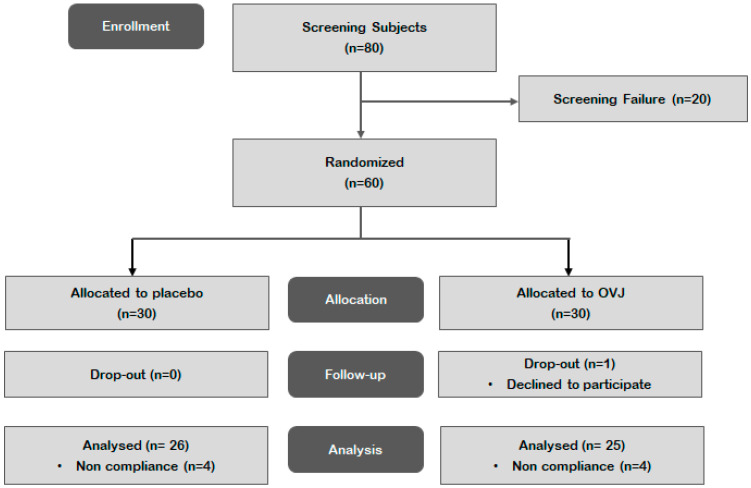
Flow chart illustrating the screening, enrollment, assignment, and follow-up of study participants for per protocol (PP) analysis.

We recruited 80 participants, enrolling the 60 who met the inclusion criteria, and randomly assigned them to two groups. A total of 59 participants completed the study. Among them, eight did not meet the intake compliance criteria (<80% treatment compliance), and one dropped out owing to withdrawal of consent during the study, thus being excluded from the analysis. Therefore, 26 and 25 participants in the placebo and OVJ groups, respectively, were included in the data analysis (Figure 1). No side effects after consuming the test food were reported as reasons for rejection. The participants’ baseline characteristics are listed in Table 3. The two groups were well matched for age, sex distribution, smoking, and drinking. They also exhibited no significant differences in anthropometric variables and vital signs.

### 3.2. Efficacy Analysis

#### 3.2.1. Biochemical Measurements (Lipid Profile Measurement)

Table 4 shows the blood lipid profile indicators (i.e., TC, TG, HDL-Cl, LDL-C, ApoB, and VLDL) of the placebo and OVJ groups at baseline and 4 weeks. The extent of change at 4 weeks relative to baseline was also presented. Statistically significant decreases in TC (Δ0.45 vs. Δ−0.40, *p* = 0.0252), TG (Δ1.70 vs. Δ−0.78, *p* = 0.0428), LDL (Δ0.25 vs. Δ−0.41, *p* = 0.0435), and ApoB (Δ0.19 vs. Δ−0.32, *p* = 0.0043) were noted in the OVJ group compared with those in the placebo group. However, HDL and VLDL did not yield statistically significant results upon comparing the OVJ and placebo groups. At baseline, no significant difference existed between the OVJ and placebo groups.

#### 3.2.2. Biochemical Measurements (Antioxidant Measurements)

Table 5 shows the antioxidant indicators (i.e., CAT, MDA, SOD, GSH, and Ox LDL) of the placebo and OVJ groups at baseline and 4 weeks. On comparing the extent of change between the OVJ and placebo groups, the OVJ group exhibited a statistically significant increase in SOD (Δ−0.15 vs. Δ0.10, *p* = 0.0440). Furthermore, GSH (Δ0.86 vs. Δ2.13, *p* = 0.0547) tended to increase in the OVJ group, though not statistically significantly. However, CAT, MDA, and Ox LDL did not exhibit statistically significant results. At baseline, no significant difference existed between the OVJ and placebo groups. 

#### 3.2.3. Biochemical Measurements (Blood Circulation-Related Measurements)

Table 6 shows the blood circulation-related indicators (i.e., e-NOS, thromboxane B2, PT, aPTT, and systolic and diastolic blood viscosities) of the placebo and OVJ groups at baseline and 4 weeks. Evidently, e-NOS, thromboxane B2, PT, and aPTT did not yield statistically significant results. Systolic (Δ0.05 vs. Δ−0.28, *p* = 0.0264) and diastolic (Δ0.25 vs. Δ−0.73, *p* = 0.0183) blood viscosities significantly decreased in the OVJ group.

### 3.3. Microbiome Analysis

#### 3.3.1. Analysis of Microbiome Diversity and OTU Classification

**Figure 2:**
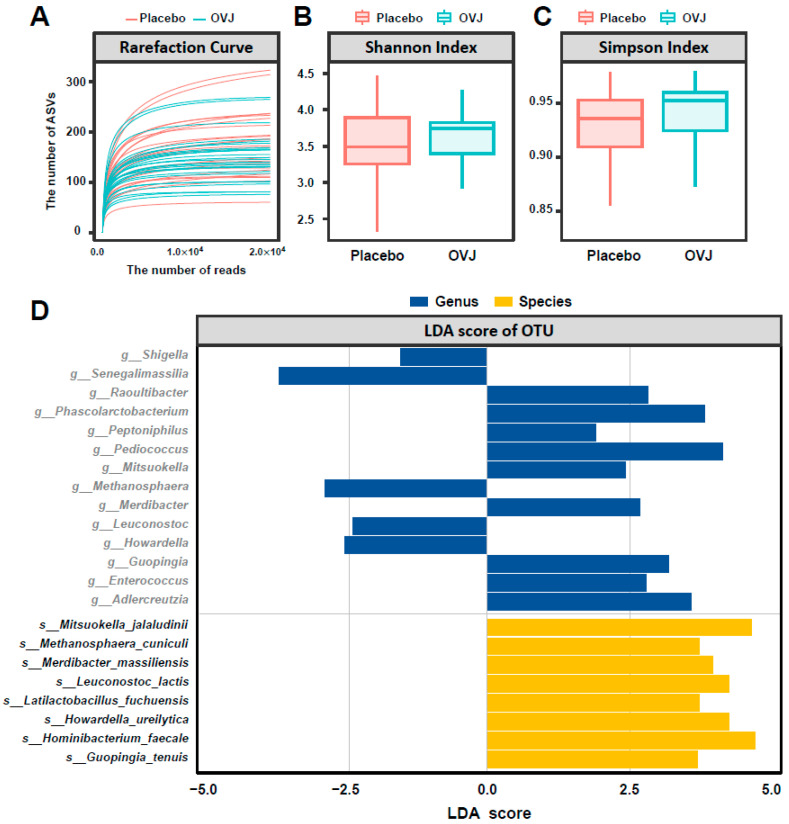
Microbiome analysis results. The graphs show the (**A**) rarefaction curve, (**B**) Shannon index, (**C**) Simpson index, and (**D**) LDA score of OTU increased or decreased strains in the OVJ and placebo groups after 4 weeks of intervention.

We rarefied samples to a sufficient sequencing depth by plotting rarefaction curves to check for saturation trends (Figure 2A). The alpha diversity index was calculated to assess the diversity of microorganisms distributed between the groups after the intervention (Figure 2B,C). Specifically, the Shannon index, a measure of alpha diversity used to quantify species diversity within a community, and the Simpson index, which indicates the probability that two randomly selected individuals from a group belong to the same species, were quantified. No significant difference in diversity was noted between the two groups after the intervention (Figure 2B,C). 

Thereafter, a linear discriminant analysis (LDA) was used to identify differentially abundant species between groups (Figure 2D). As a result, 14 strains exhibited significant differences between the OVJ and placebo groups at the genus level, while eight strains yielded significant differences at the species level. In particular, in the OVJ group, the following strains significantly increased compared with those in the placebo group: *Methanosphaera_cuniculi* (LDA score = 3.74, *p* = 0.0127), *Mitsuokella_jalaludinii* (LDA score = 4.65, *p* = 0.0160), *Latilactobacillus_fuchuensis* (LDA score = 3.74, *p* = 0.0168), *Merdibacter_massiliensis* (LDA score = 3.98, *p* = 0.0223), *Leuconostoc_lactis* (LDA score = 4.25, *p* = 0.0226), *Guopingia_tenuis* (LDA score = 3.7, *p* = 0.0323), *Hominibacterium_faecale* (LDA score = 4.71, *p* = 0.0374), and *Howardella_ureilytica* (LDA score = 4.25, *p* = 0.0421). 

#### 3.3.2. Functional Profiling Using the KEGG Pathway

**Figure 3:**
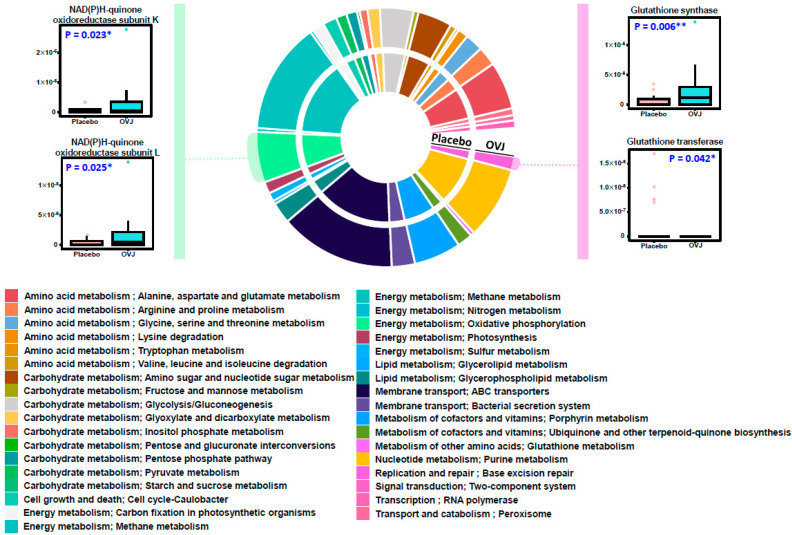
Functional profiling results based on the KEGG database. The central pie chart shows the proportion of each KEGG category, and the box plots on the side represent the KO identifiers of both groups. Statistical difference is shown by * and **, indicating *p* < 0.05 and *p* < 0.01, respectively.

Based on the above results, an analysis was conducted using the PICRUSt2 and KEGG databases to predict changes in the function of intestinal microorganisms (Figure 3). Evidently, 240 Kegg Orthology (KO) identifiers significantly differed between the OVJ and placebo groups. Among the significant KO identifiers, four were included in the oxidative phosphorylation and GSH metabolism categories.

## 4. Discussion

In this study, we conducted a clinical trial on organic vegetable mixed juice to determine the human efficacy of OVJ in improving blood circulation. The primary goal of this study was to confirm the effects of vegetable juice mixed with various vegetables, and the secondary goal was to confirm these effects by measuring indicators related to blood lipid profile, antioxidants, and blood circulation improvement. As a result, this clinical study confirmed significant changes in indicators related to blood lipid profile, antioxidants, and blood circulation improvement when juice mixed with various vegetables was consumed for four weeks. A significant increase in the antioxidant biomarker SOD and a tendency for GSH to increase were observed. It is known that lipid peroxides are hydrolyzed when the activity of antioxidant enzymes such as SOD, GSH, and CAT increases [31]. This suggests that increased antioxidant activity may have an effect on alleviating blood lipid profile, including LDL, and that OVJ consumption causes antioxidant effects, suggesting that changes in lipid composition occur due to the antioxidant effect. Additionally, improvement in blood lipid profile showed statistically significant improvements in TC, TG, LDL, and ApoB. The critical point is that this not only shows the effect of OVJ on blood lipids but also improves antioxidant markers at the same time. Additionally, SBV and DBV showed significant improvement after OVJ consumption. Blood viscosity has been reported to be closely related to cardiovascular diseases, including atherosclerosis [32,33]. Since blood viscosity is known to be influenced by blood lipid components such as LDL and HDL, it is believed that the reduction in blood lipids evaluated in this study affected the improvement of blood viscosity. Therefore, it is assumed that the significant improvement in antioxidant capacity, blood lipids, and viscosity in this study influenced each other by improving each indicator.

OVJ, used as a test juice in this study, is a mixture of various organic vegetables such as tomatoes and carrots. Because this is not a single juice but a mixed juice comprising various organic vegetables, it is presumed that the combined effects of the ingredients of each vegetable appear. Lycopene contained in tomatoes, the main ingredient of OVJ, is well known to help with fat breakdown, antioxidants, and blood circulation [34,35]. In addition, carrots, another vegetable, contain carotenoids such as β-carotene, α-carotene, lutein, and lycopene, and are well known to have cholesterol absorption and lipid decomposition effects. It is known that beta-carotene not only acts as a powerful antioxidant but also helps improve endothelial function and improves aortic cardiovascular disease [36]. Green leafy vegetables provide adequate amounts of various vitamins and minerals for humans. They contain vitamins such as pro-vitamin A, ascorbic acid, riboflavin, and folic acid and minerals such as calcium and iron. Vitamin C is the main water-soluble antioxidant in the human body [37]. Additionally, green leafy vegetables have traditionally been recognized as a good source of dietary fiber [38]. Fiber reduces cholesterol levels by preventing the reabsorption of cholesterol, which is produced in the body to aid in the digestion of fat [39]. In another previous study, an animal experiment, dietary supplements containing antioxidant-rich leafy vegetable mix (LVM) were found to be useful in protecting cells from lipid peroxidation and oxidative DNA damage in C57BL/6J mice fed a high-fat and high-cholesterol diet for 4 weeks [40]. Through this, it is assumed that leafy vegetables, such as the organic vegetable juice contained in OVJ, have a beneficial effect on improving plasma lipid profile, tissue lipid peroxidation, and vascular health against oxidative DNA damage. Therefore, it is assumed that the combined effect of various ingredients contained in OVJ affects lipid metabolism and antioxidants, improving blood circulation. This study suggests the positive impact of consuming vegetable juice mixed with various vegetables on the circulatory system.

To determine changes in gut microbiota due to OVJ consumption, metagenomic sequencing was performed. Significant differences were found between groups in the comparison between 14 genera and 8 species. Interestingly, the OVJ group showed a significant decrease in the genus Shigella, which produces trimethylamine (TMA) and increases plasma TMA oxide (TMAO) levels [41]. TMAO, a metabolite produced by intestinal microbiota, is known to be involved in the development of cardiovascular diseases such as coronary artery disease and atherosclerosis [42,43]. This suggests that OVJ may have influenced gut microbiota to improve blood flow. Our results suggest that vegetable juice consumption may help mitigate the risk of cardiovascular disease by modulating the gut microbiota involved in TMAO production. This is likely related to the improvements in blood lipid profile and blood circulation parameters assessed in this study. Moreover, eight bacteria increased in OVJ have functionality-producing beneficial substances such as SCFA, oligosaccharides, and exopolysaccharides [44,45,46,47,48]. SCFA-producing bacteria are known to maintain a healthy intestinal environment and mitigate the risk of cardiovascular disease by regulating cholesterol and lipid metabolism [49,50,51]. These results suggest that OVJ may contribute to improved blood circulation, potentially benefiting cardiovascular health, through its impact on gut microbiota. Although our study did not directly measure changes in TMAO and SCFA levels, incorporating measurements of these metabolites in further research would provide a more detailed understanding of how vegetable beverage consumption influences gut microbiota and its associated metabolic outcomes.

Functional profiling was also conducted to estimate the functional changes in the gut microbiome. Increased function was predicted to be related to glutathione synthase and oxidative phosphorylation. Both functions are known to play an important role in the antioxidant system. In our clinical results, the significant increase in SOD and the tendency for GSH to increase appear similar to this prediction [52,53]. To summarize, changes in microbial composition from OVJ consumption may affect blood circulation, lipid metabolism, and antioxidant activity in participants in this study. Although no statistically significant difference could be identified in the diversity of the intestinal microbiome, changes in composition, such as the reduction in specific harmful bacteria and functional predictions that positively affect the antioxidant system, affect the blood lipid profile, and antioxidant indicators ultimately affect blood lipid levels. It is assumed that it helped improve the circulation system.

## 5. Conclusions

Our clinical study was an exciting example of how the consumption of various mixed vegetable juices has a simultaneous positive effect on blood lipid profile, antioxidant capacity, and improvement of blood circulation. However, additional experimental verification will be needed to understand the relationship between blood biomarkers (blood lipids, antioxidants, and blood circulation improvement) and changes in intestinal microbial composition and to confirm the mechanism of action.

## Figures and Tables

**Table 1:** Nutrient composition of organic vegetable mixed juice (OVJ).

Ingredients	OVJ	Daily Value
Calories (kcal/200 mL)	106.69	-
Carbohydrate (g/200 mL)	21.69	324 g
Sugars (g/200 mL)	19.60	100 g
Crude protein (g/200 mL)	2.37	55 g
Crude fat (g/200 mL)	1.16	54 g
Saturated fat (g/200 mL)	0.27	15 g
Trans fat (g/200 mL)	0.00	-
Cholesterol (mg/200 mL)	0.00	300 mg
Na (mg/200 mL)	112.40	2000 mg
Fiber (g/200 mL)	4.69	25 g

**Table 2:** Functional ingredients of organic vegetable mixed juice (OVJ).

Functional Ingredients	Content (μg/200 mL)
Lycopene	12,929.22
β-Carotene	22,862.91

**Table 3:** Participant baseline characteristics.

Variables	Placebo (*n* = 26)	OVJ (*n* = 25)	*p*-Value
Sex (*n*, female/male)	15/11	14/11	>0.99 (C)
Smoker (Y/N)	3/23	4/21	0.7030 (F)
Drinker (Y/N)	18/8	21/4	0.3613 (C)
Age (years)	37.08 ± 1.88	36.72 ± 1.71	0.8889 (W)
Weight (kg)	74.08 ± 2.23	72.4 ± 2.80	0.6408 (T)
BMI (kg/m^2^)	26.45 ± 0.48	25.64 ± 0.60	0.2937 (T)
SBP (mmHg)	124.69 ± 2.21	127.28 ± 2.26	0.4168 (T)
DBP (mmHg)	77.62 ± 1.90	76.40 ± 1.80	0.6441 (W)

The data points correspond to the mean ± SEM.

**Table 4:** Comparison of serum lipid profile measurements between the placebo and OVJ groups.

Variables	Placebo (*n* = 26)			OVJ (*n* = 25)			*p*-Value ^a^	*p*-Value ^b^
	Baseline	4 Weeks	Change	Baseline	4 Weeks	Change		
Total cholesterol (mmol/L)	11.99 ± 0.06	12.44 ± 0.07	0.45 ± 0.05	12.14 ± 0.09	11.74 ± 0.09	−0.40 ± 0.05	0.0252 (T) *	0.7898 (T)
Triglyceride (mmol/L)	6.69 ± 0.14	8.40 ± 0.34	1.70 ± 0.25	6.86 ± 0.18	6.09 ± 0.15	−0.78 ± 0.16	0.0428 (W) *	0.7702 (W)
HDL cholesterol (mmol/L)	3.54 ± 0.04	3.31 ± 0.04	−0.23 ± 0.02	3.44 ± 0.03	3.47 ± 0.03	0.03 ± 0.02	0.5457 (W)	0.7069 (W)
LDL cholesterol (mmol/L)	7.40 ± 0.05	7.65 ± 0.06	0.25 ± 0.05	7.56 ± 0.07	7.15 ± 0.08	−0.41 ± 0.04	0.0435 (T) *	0.7277 (T)
ApoB (mmol/L)	5.10 ± 0.03	5.29 ± 0.03	0.19 ± 0.02	5.22 ± 0.05	4.91 ± 0.05	−0.32 ± 0.02	0.0043 (T) **	0.6665 (W)
VLDL (mmol/L)	1.06 ± 0.02	1.48 ± 0.05	0.43 ± 0.04	1.08 ± 0.03	1.12 ± 0.02	0.04 ± 0.02	0.1867 (W)	0.7483 (W)

The data points correspond to the mean ± SEM. ^a^ Change differences between the placebo and OVJ groups. The *p*-value was obtained from an independent *t*-test or Mann–Whitney U test. ^b^ Differences between the placebo and OVJ groups at baseline. The *p*-value was obtained from an independent *t*-test or Mann–Whitney U test. Statistical difference is shown by * and **, indicating *p* < 0.05 and *p* < 0.01, respectively.

**Table 5:** Comparison of serum antioxidant measurements between the placebo and OVJ groups.

Variables	Placebo (*n* = 26)			OVJ (*n* = 25)			*p*-Value ^a^	*p*-Value ^b^
	Baseline	4 Weeks	Change	Baseline	4 Weeks	Change		
Catalase (ng/mL)	1829.77 ± 94.40	2085.36 ± 107.67	255.59 ± 22.69	2102.98 ± 144.92	2252.67 ± 134.95	149.69 ± 43.67	0.6707 (T)	0.1218 (T)
Malondialdehyde (ng/mL)	0.43 ± 0.01	0.47 ± 0.02	0.04 ± 0.01	0.52 ± 0.02	0.57 ± 0.02	0.04 ± 0.01	0.6839 (W)	0.3201 (W)
SOD (ng/mL)	2.19 ± 0.20	2.05 ± 0.18	−0.15 ± 0.02	1.97 ± 0.12	2.07 ± 0.15	0.10 ± 0.02	0.0440 (T) *	0.5719 (W)
GSH (μg/mL)	16.2 ± 1.32	17.06 ± 1.55	0.86 ± 0.10	14.65 ± 1.02	16.77 ± 1.16	2.13 ± 0.08	0.0547 (W)	0.2622 (W)
Ox LDL (ng/mL)	116.69 ± 6.69	104.55 ± 6.06	−12.13 ± 1.54	123.13 ± 10.75	109.43 ± 8.47	−13.7 ± 2.01	0.9029 (W)	>0.99 (W)

The data points correspond to the mean ± SEM. ^a^ Change differences between the placebo and OVJ groups. The *p*-value was obtained from an independent *t*-test or Mann–Whitney U test. ^b^ Differences between the placebo and OVJ groups at baseline. The *p*-value was obtained from an independent *t*-test or Mann–Whitney U test. Statistical difference is shown by *, indicating *p* < 0.05.

**Table 6:** Comparison of serum blood circulation-related measurements between the placebo and OVJ groups.

Variables	Placebo (*n* = 26)			OVJ (*n* = 25)			*p*-Value ^a^	*p*-Value ^b^
	Baseline	4 Weeks	Change	Baseline	4 Weeks	Change		
e-NOS (ng/mL)	0.64 ± 0.49	0.69 ± 0.53	0.05 ± 0.31	0.82 ± 0.02	0.86 ± 0.02	0.04 ± 0.01	0.7437 (W)	0.1617 (W)
Thromboxane B2 (ng/mL)	8.80 ± 2.52	9.94 ± 2.81	1.15 ± 0.47	8.60 ± 2.37	15.13 ± 7.09	6.54 ± 1.51	0.8728 (W)	0.6579 (W)
PT (s)	13.36 ± 0.12	13.38 ± 0.11	0.02 ± 0.02	13.36 ± 0.10	13.47 ± 0.10	0.11 ± 0.02	0.5543 (T)	0.9713 (T)
aPTT (s)	34.77 ± 0.57	35.57 ± 0.48	0.79 ± 0.08	35.78 ± 0.67	36.90 ± 0.76	1.12 ± 0.08	0.3460 (T)	0.2600 (T)
SBV (cP)	4.94 ± 0.16	4.99 ± 0.15	0.05 ± 0.02	5.21 ± 0.16	4.92 ± 0.15	−0.28 ± 0.02	0.0264 (T) *	0.2454 (T)
DBV (cP)	14.92 ± 0.41	15.00 ± 0.42	0.25 ± 0.06	15.43 ± 0.41	14.7 ± 0.44	−0.73 ± 0.06	0.0183 (T) *	0.2440 (T)

The data points correspond to the mean ± SEM. ^a^ Change differences between the placebo and OVJ groups. The *p*-value was obtained from an independent *t*-test or Mann–Whitney U test. ^b^ Differences between the placebo and OVJ groups at baseline. The *p*-value was obtained from an independent *t*-test or Mann–Whitney U test. Statistical difference is shown by *, indicating *p* < 0.05.

## Data Availability

The data analyzed during the present study are available from the corresponding author, further inquiries can be directed to the corresponding author.
